# “Like Taking Part in Star Wars”: Qualitative Study Using Thematic Analysis to Explore the Acceptability and Experiences of Older Adults Participating in Remote Longitudinal Sleep and Dementia Research

**DOI:** 10.2196/88094

**Published:** 2026-08-18

**Authors:** Bijetri Biswas, Victoria Grace Gabb, Jonathan Blackman, Hamish Morrison, Elizabeth Coulthard, Anne Roudaut

**Affiliations:** 1 Faculty of Science and Engineering, University of Bristol, Bristol, England, United Kingdom; 2 ReMemBr Group, North Bristol NHS Trust, Bristol, England, United Kingdom; 3 Bristol Medical School, University of Bristol, Second Floor, Learning & Research Building, Southmead Hospital, Bristol, BS10 5NB, United Kingdom, 44 0117 928 9000; 4 NIHR Bristol Biomedical Research Centre, Bristol, England, United Kingdom

**Keywords:** dementia, mild cognitive impairment, sleep, technology, digital health, wearables, acceptability, engagement, usability, patient-centered research

## Abstract

**Background:**

Sleep disturbance is a common symptom of and potential risk factor for neurodegeneration and dementia. Remote monitoring technologies and increasing digital literacy offer promise for monitoring symptoms and treatment responses via sleep and cognitive assessments from patients’ homes. However, the acceptability of remote sleep and circadian research in older adults with and without cognitive impairment is not known.

**Objective:**

This study aimed to explore and describe the barriers, facilitators, and user experience of older adults participating in longitudinal sleep and dementia research using remote monitoring technologies.

**Methods:**

Older adults with mild cognitive impairment (MCI) or dementia due to probable Alzheimer disease or Lewy body disease and age-matched controls participated in an 8-week remote study involving multimodal assessments of sleep and cognition, including actigraphy, wireless electroencephalography, web-based cognitive tasks, and serial saliva samples. Participants were asked for feedback via questionnaires during the study at 2 time points and purposively invited to complete end-of-study interviews about their experiences. The Capability, Opportunity, Motivation–Behavior model of behavior change, and the extended Unified Theory of Acceptance and Use of Technology, were used to guide questionnaire and interview topic guide development. Inductive reflexive thematic analysis was undertaken, with components from the models used as sensitizing concepts.

**Results:**

A total of 14 participants (9 with MCI or dementia, 5 controls) completed end-of-study interviews, and 28 participants (9 with MCI or dementia, 19 controls) completed questionnaires. Six key themes were identified: (1) “Perceived value as motivation,” (2) “Trust and simplicity as cornerstones in user experience,” (3) “Adjusting to study participation over time,” (4) “Adherence, accuracy, and getting it right,” (5) “Social support as a facilitator and a barrier,” and (6) “Reflections, realities, and uncertainties around sleep.”

**Conclusions:**

Older adults with and without cognitive impairment were motivated to engage in longitudinal remote sleep research and provide good quality data. Acceptability was related to burden, usability, and reliability of devices, having sufficient support, and ability to build study tasks into a routine. In repeated cognitive tasks, varying task content and difficulty, and allowing flexibility in timing may avoid fatigue and frustration. Future studies should aim to identify effective strategies for recruiting diverse populations, particularly those with limited technology experience or from underserved communities, to ensure equitable participation and representation in research. Providing education on the importance of sleep for brain health and technology use may be beneficial.

## Introduction

Sleep changes throughout the life span. As we enter older adulthood, sleep generally becomes shorter, “lighter” (less time spent in deep or slow-wave sleep), and more fragmented than in midlife [1,2]. While these changes are considered normal, nearly half of older adults report sleep complaints such as poor sleep quality, insomnia, and daytime fatigue [3]. Rather than a decreased *need* for sleep, it has been argued that older adults have a reduced *ability* to sleep due to the normative changes to sleep architecture alongside the increasing prevalence of age-related health conditions impacting sleep, including nocturia, sleep apnea, heart disease, and polypharmacy [4,5].

Sleep disturbance is also associated with cognitive impairment and neurodegeneration. In Alzheimer disease (AD), sleep disturbances often reflect an exaggeration of the changes observed in healthy aging [6]. In contrast, Lewy body disease (LBD) is often preceded by loss of atonia during rapid eye movement (REM) sleep (REM sleep behavior disorder), and patients with LBD tend to report poor subjective sleep quality [7,8]. The relationship between sleep and neurodegeneration is considered bidirectional, with short sleep duration and sleep disorders in midlife associated with increased dementia risk [9,10]. Proposed mechanisms linking sleep and dementia include impaired cerebral clearance of metabolic waste products such as amyloid beta [11], increased neuroinflammation [12], and worse cardiovascular health [13]. While data from epidemiological and animal studies increasingly support sleep as a modifiable risk factor for dementia, prospective studies and high-quality clinical trials are needed to better understand the underlying mechanisms and identify which components of sleep represent optimal therapeutic targets [14,15].

To date, sleep research in individuals with dementia or mild cognitive impairment (MCI) has largely used self-report or polysomnography [16]. Self-report measures are easy to administer and capture subjective sleep quality, but not sleep microarchitecture or sleep staging, and are poorly aligned with objective sleep parameters [17]. Polysomnography provides rich objective data but does not capture naturalistic sleep and lacks scalability due to the resources required [18]. Sleep assessments offering deep sleep profiling which are pragmatic and suitable for future large-scale clinical trials and cohort studies in patients with cognitive impairment are warranted.

Remote monitoring technologies (RMTs) including wearables such as electroencephalography (EEG) sleep headbands and actigraphy, nearables such as mattress sensors, digital health platforms, and apps could offer pragmatic and scalable alternatives able to provide rich multimodal sleep data over a longer duration than polysomnography [19,20]. Alongside sleep measures, other digital health technologies and remote study tasks could allow more data to be collected in the home, enabling researchers to collect more naturalistic high-frequency data and reduce study burden. For example, home-based saliva testing could capture naturalistic cortisol awakening response and dim-light melatonin onset [21], and remote cognitive testing and questionnaires could reduce the need for lengthy traditional clinic-based assessments [22,23]. In-depth, multimodal longitudinal sleep and cognitive profiling from home might better capture intraindividual variation over time and offer a pragmatic and decentralized way to more closely monitor the safety and efficacy of new sleep interventions [24]. However, such research designs represent a considerable shift from clinical trial paradigms often adopted in dementia research, and their acceptability in this population is not yet understood.

Older adults, including people living with MCI or dementia, increasingly use the internet and digital health technologies to support their daily lives [25,26]. However, large numbers of older adults still have limited or no access to technology [27], and technology adoption can be stressful for older adults and individuals with cognitive difficulties [28]. Cognitive impairment may directly affect how someone interacts with technology or their confidence or ability to learn new processes [29]. Research on the usability and acceptability of technology in older adults living with MCI or dementia has so far focused on technology designed to be incorporated into daily life for the patient’s benefit: for navigation, safety monitoring, to support everyday functioning, or for entertainment and leisure purposes [30,31]. Using technology for research purposes, rather than personal benefit, may be evaluated differently [32]. One recent study identified promising usability of apps and wearables by participants with AD in research, particularly for passive monitoring devices, but focused largely on “problem rates” rather than acceptability or overall experiences, and all participants had support from a study partner [20]. There is currently an unmet need for qualitative research exploring whether remote sleep research in older adults with and at-risk of dementia is acceptable and how study design could be optimized for future clinical trials.

This study aimed to describe the experiences of older adults who participated in the RESTED (Remote Evaluation of Sleep to Enhance Understanding of Early Dementia) study, a predominantly remote longitudinal dementia research study using multimodal sleep and cognitive data collection supported by digital health technologies. Specifically, we focused on barriers and facilitators to study recruitment and retention and the acceptability and user experience of remote sleep and cognitive assessments.

## Methods

### Overview

The full study protocol has been published online [33]. This analysis is part of a larger observational prospective cohort study, the RESTED study, which was designed to examine the acceptability and feasibility of remote sleep monitoring in older adults with and without MCI and dementia [33,34] and compare sleep differences between the cohorts [8,35]. Here, we describe a qualitative study using thematic analysis to explore the acceptability and user experience of remote sleep and dementia research. This study is reported in accordance with the Standards for Reporting Qualitative Research [36] (Multimedia Appendix 1).

### Participants

All participants were aged ≥50 years and either met established clinical criteria for MCI or dementia due to probable AD or LBD or were age-matched cognitively healthy controls. Participants who scored ≤11 on the Montreal Cognitive Assessment [37] during screening or had untreated comorbidities unrelated to an MCI or dementia diagnosis that could significantly interfere with sleep were excluded. Participants were identified through cognitive and movement disorders clinics at North Bristol NHS Trust and research volunteer databases.

### Study Assessments

Participants completed baseline clinic assessments on sleep, cognition, and mood [33] in clinic, followed by 8 weeks of remote data collection. Objective sleep was assessed using a wrist-worn actigraphy device (*Axivity AX3*) throughout and complemented by daily digital sleep diaries collected via an app (*MyDignio*) installed on a mobile or tablet device. Twice weekly, participants completed 3 consecutive remote unsupervised cognitive assessments via an online platform (*Cognitron*): choice reaction time, forward digit span, and self-ordered search.

For 7 days during the main study period (the “intensive week”), additional data were collected. Objective sleep was measured using a wireless EEG headband (*Dreem 2*) [38,39]. Cognition was assessed with daily unsupervised cognitive assessments (*Cognitron*) and 2 supervised verbal memory tests administered remotely via videoconferencing with the research team (*Microsoft Teams*) across 2 evenings (to learn a word list) and the following mornings (to test recall and recognition). Circadian rhythms were measured using serial saliva samples collected at home to measure dim-light melatonin onset (across 1 evening) and cortisol awakening response (across 1 morning). Participants with no diagnosis of sleep apnea or recent sleep apnea assessment were also asked to complete 2 consecutive nights of overnight pulse oximetry (*Nonin 3150 WristOx2*).

### Qualitative Data Collection

**Figure 1.**
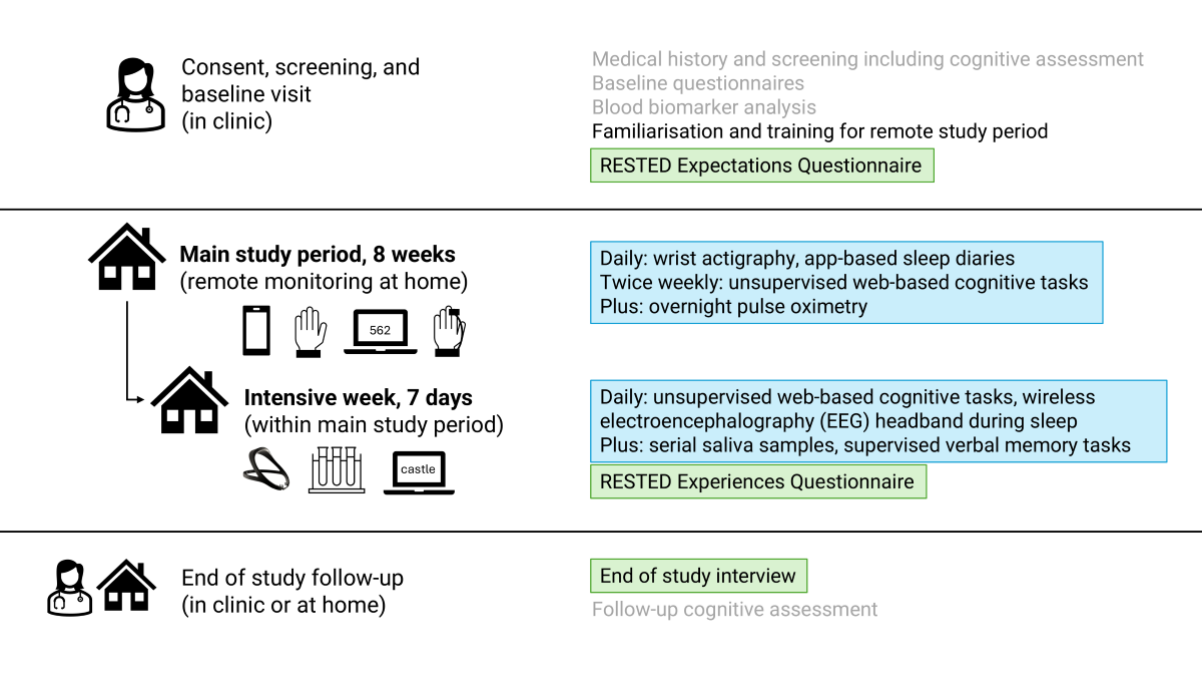
Overview of data collection in the RESTED study. Remote study tasks were administered across 8 weeks and are highlighted (blue). Qualitative data were collected during the main study period through questionnaires and an optional end-of-study interview (green). Study procedures in black text were the primary focus of this acceptability analysis. RESTED: Remote Evaluation of Sleep to Enhance Understanding of Early Dementia.

Figure 1 illustrates the study design and highlights when and how qualitative data were collected. The present thematic analysis incorporated data collected predominantly during the end-of-study semistructured interviews, as well as through questionnaires before starting the main period of data collection and following the intensive week.

#### End-of-Study Interviews

Semistructured interviews were conducted online and audio-recorded with verbal consent. Interviews typically lasted 30-60 minutes. The interview topic guide was developed based on the Capability, Opportunity, Motivation–Behavior (COM-B) [40] model (Multimedia Appendix 1). Topics included experiences of recruitment, study devices, remote contact with researchers, overall study impressions, and general feedback. Participants were explicitly asked to recall both positive and negative aspects of the study, as well as what might be improved in future research designs.

Maximal variation sampling, a form of purposive sampling, was used to nonrandomly invite participants to end-of-study interviews, based on characteristics considered relevant to acceptability of remote sleep research: age, sex, educational background, employment status, subjective sleep quality, and cohort (AD, LBD, or controls). Participants who withdrew from the study or specifically contacted the research team with study feedback were also invited to participate in an interview.

#### Questionnaires

To supplement the interview data, 2 questionnaires were introduced following an ethically approved study amendment during the recruitment period: the RESTED Expectations and RESTED Experiences questionnaires (Multimedia Appendix 1). Participants joining the study or who had not yet completed their intensive week of assessments were encouraged to complete the questionnaires at the relevant time points. Participants who had completed their intensive week prior to the amendment implementation were not asked to retrospectively complete questionnaires.

Both questionnaires were developed with reference to the COM-B model and the extended Unified Theory of Acceptance and Use of Technology (UTAUT-2) model [32] and aimed to provide additional insight on participants’ perspectives prior to and during active engagement in the study. The RESTED Expectations questionnaire probed motivations, first impressions of study devices, and prior experience of technology. The RESTED Experiences questionnaire focused on the intensive week of the study, which specific tasks were easier or harder to complete, and whether research participation had influenced day-to-day life or social functioning.

### Data Analysis

Reflexive thematic analysis [41] was used to analyze the interview transcripts and free-text responses from the questionnaires. Codes were generated through an inductive or “bottom-up” data-driven approach [42], with concepts from both the COM-B [40] and UTAUT-2 [32] models used as sensitizing concepts during coding and theme development to guide interpretation.

Data analysis was informed by Braun and Clarke’s [43] 6 phases of thematic analysis. Interview transcripts and free-text questionnaire responses were collated and analyzed by 2 authors (BB and VGG). The authors familiarized themselves with the dataset via independently reviewing the data and making initial notes. Preliminary codes were proposed, discussed, and iteratively combined into broader initial themes before discussion and review with the wider study team. BB conducted a final check to ensure that all relevant codes were captured and themes were refined to reflect participants’ experiences accurately. Findings are presented with detailed descriptions of the themes, alongside illustrative quotes extracted from the dataset.

### Reflexivity

Data were collected and analyzed by the joint first authors (BB and VGG). BB has a background in digital health technology and VGG predominantly works in sleep and dementia research, with a background in health research and neuroscience. As BB was primarily involved in the qualitative elements of the study, she was not known to participants prior to interviews, whereas as the lead research assistant and main point of contact for participants during the study, VGG was known to all research participants. This familiarity was advantageous in fostering openness and trust during interviews but required ongoing reflexive awareness of how prior interactions might influence participants’ responses and the interpretation of their narratives. The authors made conscious efforts to discuss and challenge assumptions and perspectives that may have influenced their interpretation of the data.

Steps were taken to diminish the distance or power imbalance in the “‘researcher-researched” relationship [44]. Interviews were conducted remotely, with participants taking part from home at a time of their choosing, with partners present where participants felt more comfortable with this setup [45]. Within the interview, participants were actively encouraged to share both positive and negative experiences to help the research team learn and share their advice for future research. The semistructured interview design and free-text responses in the questionnaire allowed participants to guide conversations or comments toward what they perceived as important or relevant. These reflexive, participant-centered strategies aimed to strengthen the study’s credibility and trustworthiness by promoting openness, inclusion, and coconstruction of meaning between researchers and participants.

### Ethical Considerations

This study was approved by the Health Research Authority and Yorkshire and the Humber—Bradford Leeds Research Ethics Committee (reference 21/YH/0177) and conducted in accordance with Good Clinical Practice and the Declaration of Helsinki. All participants received detailed information about the study and provided written informed consent before participation. Participants were reimbursed for travel expenses incurred during the study but not for their study participation. Data collected were pseudonymized and identifiable information was redacted prior to analysis. To protect participant anonymity, particularly given the sample size and the sensitive nature of the topic, we chose not to associate quotes with individual identifiers. Instead, our analysis focused on shared thematic patterns, rather than individual-level variation. Quotes were selected to represent the breadth and diversity of perspectives expressed during the interviews.

## Results

### Participants

A total of 41 participants were recruited into the RESTED study. One participant withdrew immediately after completing baseline assessments due to personal circumstances and perceived study burden and declined an interview. The remaining 40 participants completed the full study period. Full feasibility metrics (eg, adherence to study protocol) are presented in detail elsewhere [33]. Overall, 32 (80%) participants provided data for the acceptability study through completing the end-of-study interview and/or questionnaires (Table 1). Participants were mostly male, retired, and reported poor sleep quality.

**Table 1.** Baseline characteristics for participants included in the acceptability analysis.

Characteristics of participants	MCI^a^ or dementia (n*=*13)	Controls (n*=*19)		Total (N*=*32)
Age (years)**,** mean (SD)	72.4 (6.6)	70.2 (5.9)		71.1 (6.2)
Male sex**,** n (%)	10 (77)	15 (79)		25 (78)
Employment status**,** n (%)				
Full or part-time employment	5 (26)	1 (8)		6 (19)
Retired	14 (74)	12 (92)		26 (81)
Continued education past secondary school (O-level or GCSE^b^), n (%)	15 (79)	9 (69)		24 (75)
MoCA^c^ at baseline, mean (SD)	23.6 (4.4)	27.1 (1.5)		25.7 (3.4)
PSQI^d^ at baseline, mean (SD)	5.9 (2.7)	6.0 (4.0)		6.0 (3.5)

a MCI: mild cognitive impairment.

b GCSE: General Certificate of Secondary Education.

c MoCA: Montreal Cognitive Assessment.

d PSQI: Pittsburgh Sleep Quality Index.

A total of 14 participants completed end-of-study interviews (5 participants with amnestic MCI or dementia due to AD, 4 participants with MCI or dementia due to LBD, and 5 controls). In total, 28 participants (4 participants with amnestic MCI or dementia due to AD, 5 participants with MCI or dementia due to LBD, and 19 controls) completed at least one of the questionnaires. Diagnoses of patients with MCI or dementia included in the qualitative study included AD dementia (n=2), amnestic MCI or MCI due to AD (n=3), primary progressive aphasia due to probable AD (n=1), MCI due to LBD (n=4), and LBD dementia (n=3). The majority of participants reported using technology (smartphones, smartwatches, computers, or tablets) multiple times per day (18/25, 72%) but had not previously used any wearable devices to monitor their sleep (22/25, 88%).

### Summary of Themes

The thematic analysis resulted in five major themes around barriers and facilitators to engaging in remote longitudinal sleep and cognitive research: (1) *Perceived value as motivation;* (2) *Trust and simplicity as cornerstones in user experience;* (3) *Adjusting to study participation over time;* (4) *Adherence, accuracy, and getting it right*; and (5) *Social support as a facilitator and a barrier*. The analysis also identified a sixth theme, *Reflections, realities, and uncertainties around sleep,* which captured older adults’ priorities, experiences, and understanding of sleep.

**Figure 2.**
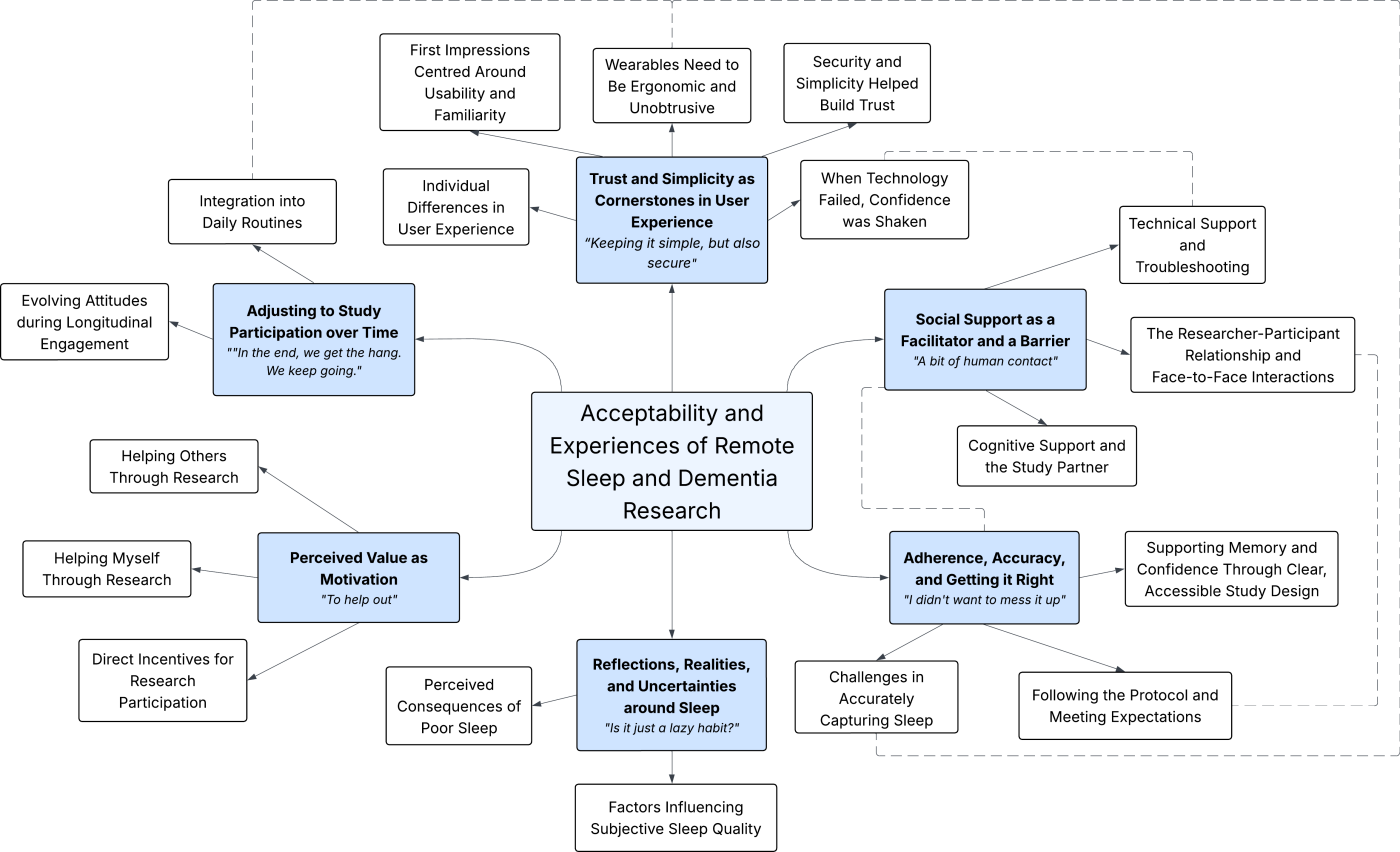
Overview of the results from the thematic analysis, capturing the key themes (in boldface) and their subthemes. Dashed lines represent key connections between themes.

An overview of the themes is presented in Figure 2, with the content of themes presented in detail in the following sections. How themes mapped onto the COM-B and UTAUT-2 models is outlined in Multimedia Appendix 1.

### Theme 1: Perceived Value as Motivation

*To help out*.

#### Theme Overview

Participants reported reflective motivations for study participation, perceiving it as helpful to themselves, family members, or society. However, some participants also identified that direct incentives, such as providing feedback on sleep or financial reimbursement, could help to motivate more people to participate in future studies.

#### “To Give Something Back”: Helping Others Through Research

Participants often cited altruistic underlying reflective motivations, recognizing that their participation would likely not directly impact themselves but might benefit others, such as family members, who might be at risk in the future:

*My two sisters and I are really quite concerned that we might get it [...] It might not help me, but it might help my family further down the line.*

Some participants cited more proximal reasons, specifically wanting to help the research team or clinic through research participation. One participant expressed how their appreciation for the service they receive from the health care and research team prompted study enrollment:

*I get good treatment from [the clinic] and it is good to give something back*.

#### “It Gave Me Something to Do”: Helping Myself Through Research

Other participants perceived more direct personal benefits to study participation. For instance, engaging in research activities such as cognitive testing was viewed as a form of cognitive stimulation that could help delay cognitive decline or help to manage symptoms:

*This sort of activity helps with my Alzheimer’s*.

Although participants were provided with personalized feedback only if they were found to have undiagnosed sleep apnea, participants also commented that study participation might indicate if they needed further investigations for their cognition or sleep. Being involved in research, and the research community, was also identified as a route to accessing potential treatments before approval.

In addition, several participants explained that the study sounded interesting or enjoyable. Research participation was generally viewed as a positive activity, helping participants to “feel useful and part of something significant.” Some participants also enjoyed having tasks to complete, suggesting that research gave them a sense of purpose or a new pastime:

*Having the structure and sort of something to be getting on with...I enjoyed it*.

Additionally, several participants noted that they took part because they had “taken part in dementia studies before” suggesting that research involvement can become habitual.

#### “Getting Something Out of It”: Direct Incentives for Research Participation

Although participants’ own motivations centered around helping themselves or others, direct incentives, such as personalized sleep or cognitive feedback or financial reimbursement to participate, were proposed as a way to facilitate recruitment to similar studies in the future:

*It would encourage people, you know, to think that they were actually getting something out of it, rather than just entirely giving*.

Personalized sleep feedback was generally considered as interesting and informative but not necessarily useful or something to act on. Unless a problem was identified, few participants intended to change their sleeping habits:

*Out of curiosity and personal interest, and again, [to] feel kind of valued that you actually, you’ve done something and you’re just getting some information that’s not any judgment or diagnosis or comments, just “this is what we found about your sleep patterns.”*

Overall, motivations to participate centered around the perception of the study as helpful to themselves or others, although direct incentives (financial or nonfinancial) could also potentially motivate study participation.

### Theme 2: Trust and Simplicity as Cornerstones in User Experience

*Keeping it simple, but also secure*.

#### Theme Overview

Simplicity and trusting devices to work as expected was central to user experience, particularly as most study devices were unfamiliar to participants. User experience was influenced by factors such as novelty, usability, comfort, technical reliability, and privacy.

#### “Like Taking Part in Star Wars”: First Impressions Centered Around Usability and Familiarity

Despite most participants reporting frequent exposure to smart technology at baseline, participants were largely unfamiliar with the specific study devices or sleep wearables more generally. Participants compared unfamiliar study devices with ones they had come across before in their personal life, such as fitness trackers, creating mental anchors that helped to contextualize their purpose:

*I don’t even have, you know, a Fitbit or anything like that normally. So no, I hadn’t [come across the technology before]. Apps obviously are all a bit new*.

Beyond novelty, participants typically focused on practicalities, initially evaluating devices by whether they appeared simple to use and comfortable to wear.

#### “You’d Have to Change the Design a Little Bit”: Wearables Need to Be Ergonomic and Unobtrusive

For the most part, participants found wearable devices tolerable and minimally intrusive to their day-to-day life or sleep:

*I just put it on and left it on and it was fine. Didn’t trouble me.* [talking about the actigraphy device]

However, several improvements were suggested around ergonomic design. The silicone strap of the actigraphy watch could catch on clothing, which could interfere with activities of daily living such as getting dressed, and prolonged use also occasionally caused mild skin irritation. Participants recommended making the device smaller, lighter, and more streamlined, and possibly with a watch face, to make it more aesthetically pleasing and functional:

*Everything else has got more streamlined—mobile phones and the rest of it. That just seemed...like wearing a miniature brick*.

Similarly, while the Dreem 2 headband was largely well-tolerated, some participants identified challenges in comfort and design. For example, the small red light near the forehead sensors, which indicated that a recording was in progress, could be seen by some participants and could disrupt sleep. Positioning the headband securely enough to get a good quality trace, without being uncomfortable, was also sometimes a challenge for participants:

*The [Dreem 2] headband was an issue because of the red light. It kept slipping down. I got a headband, you know, my own headband. Put it round. But I could still see this red light*.

Simultaneous use of multiple wearable technologies could also become uncomfortable and less acceptable compared with using 1 device alone. For example, participants with sleep apnea who were using a continuous positive airway pressure mask had some difficulties with fitting the mask and the EEG headband comfortably.

#### “It Was Perfectly Useful Tech, You Know, When It Worked”: When Technology Failed, Confidence Was Shaken

Technical reliability, or how consistently the technology performed according to participants’ expectations, was also central to user experience and acceptability. Several participants reported technical glitches, often triggered by software updates. When expectations of the technology were not met, participants became frustrated, blamed or questioned their technical or cognitive abilities, and sought support and reassurance from others:

*I got my husband to look at the computer. I’m quite computer literate. And I got him to look at it and I said, “I don’t know what I’m doing wrong” and he said, “I don’t think you’re doing anything wrong. It is a software problem.”*

Participants also sometimes experienced confusion or frustration with interacting with different apps or websites for different tasks (eg, a mobile app for the sleep diary and a website for the cognitive tests). Developing a single platform for all remote study tasks was suggested to make the study more straightforward for participants in future studies and support better user experience.

#### “I Didn’t Find Anything Intrusive”: Security and Simplicity Helped Build Trust

Privacy and data security were key to engagement with many participants emphasizing the importance of nonintrusive data collection, such as the absence of cameras which are typically present during full polysomnography, or questions about sensitive topics such as dream content:

*The idea of perhaps cameras on you, that would be horrible as well, wouldn’t it? It was that, if somebody was watching what you do when you’re asleep? Yeah, then you might do ridiculous things or not very dignified things. I’d feel that would be an invasion of privacy*.

Participants often described how study tasks were not perceived as invasive or intrusive and understood that their participation was voluntary, which fostered a sense of trust and control. Protecting data security while keeping device and software interactions straightforward was also identified as a priority:

*Getting that balance between keeping it simple, but also secure. And the participants who are of a certain age group, I guess, need that confidence and the simplicity*.

#### “This Is My Psychology. I Don’t Know if It’s Everyone’s Psychology”: Individual Differences in User Experiences

Participants often had different opinions on which components of the study were the easiest or hardest or the most or least enjoyable to complete. For example, many participants found it particularly challenging to collect saliva samples at home and reported both cognitive difficulties (eg, understanding the instructions and accurately timing sample collection) and practical difficulties (eg, fitting samples in around existing commitments and physically producing sufficient saliva):

*Those wretched saliva tests are an absolute nightmare. I have never in my life ever done a saliva test. Yeah, so parcelling out exactly what you’ve got to do, and then also realising how often you’ve got to do it, and then juggling the idea of what, how do you fit in your normal thing[s]?*

However, other participants felt that home saliva testing was straightforward or an enjoyable part of the study, highlighting how user experience can vary substantially across participants and be influenced by ease of use and personal satisfaction:

*I enjoyed the physical thing, where physical things are involved as well as just the questions and answers. Like the saliva testing and things like that*.

Limited digital literacy or confidence, due to older age or less exposure to technology, was suggested as a possible influence on participants’ experiences of whether research using RMTs was acceptable or enjoyable:

*I’d imagine somebody a lot older would probably struggle with some of that, particularly the technology side of it*.

Additionally, physical challenges could also complicate interactions with devices and study tasks. For instance, being able to respond quickly and accurately in the online cognitive tasks could be more challenging for people who may have conditions affecting their dexterity, such as arthritis or parkinsonism, introducing confounding factors. Beyond variations in preferences and perceived ease of use across participants, an individual’s experience was also not necessarily static or in line with their first impressions.

### Theme 3: Adjusting to Study Participation Over Time

*In the end, we get the hang. We keep going*.

#### Theme Overview

Perceptions of the study and individual tasks evolved over time. Being able to integrate study tasks conveniently into existing routines or commitments facilitated engagement and reduced perceptions of burden, while time-restricted tasks were a barrier. Prolonged engagement in research encouraged participants to adapt and find strategies which supported their engagement in study tasks, but repetition of challenging tasks was also linked to disengagement and study fatigue.

#### “We’re Sort of Busy People”: Integration Into Daily Routines

Being able to integrate study tasks within existing routines or commitments facilitated participant engagement and adherence. At the outset, some participants were concerned that study participation might be burdensome or disruptive to their daily lives:

*[What] I didn’t want it to do was to interfere with, you know, sort of my own life too much, and it didn’t...That was really my own concern, because we’re sort of busy people, and the last thing you want to do is, you know, be doing something where you think “I haven’t got time.”*

Being able to develop habits or routines to complete their study tasks around their usual activities helped to reduce participant burden. To minimize burden, some participants planned out the day and prepared ahead of time by placing equipment or devices required for study tasks in plain sight:

*As soon as I woke up, get a cup of coffee, do the morning, some pieces, and then it was forgotten for the rest of the day*.

Smartphones and digital tablets, compared with pen and paper assessments or personal computers, enabled greater flexibility around when and where study tasks could be completed, such as at home or at work. Passive data collection enabled by the actigraphy watch also supported participants to continue with their usual activities. In contrast, tasks that were requested at a specific time of day were more challenging to incorporate into routines and around existing commitments, such as trying to fit in cognitive tests before going to work:

*It was demanding for me, because I have commitments on nearly every day of the week, which require me to get out of the house quite early*.

Participants generally accepted the inconvenience of time-limited tasks which occurred infrequently (eg, once or twice during the study period); however, time-specific tasks that were repeated more frequently were less acceptable. When unavailable during the designated time, some participants felt stressed or skipped the tasks altogether, suggesting that flexibility in timing could influence adherence as well as study burden.

#### “I Got a Bit Competitive With It”: Evolving Attitudes During Longitudinal Engagement

Participants’ attitudes toward the study tasks often changed over the course of the study. With time and experience, participants became more confident and were able to complete various tasks at differing time intervals without too much difficulty:

*I think once you started getting the hang of putting it all together, it was easier*.

Throughout the study, participants often took initiative to make it easier to complete study tasks or improve their performance, suggesting that repeated exposure to the same tasks created a learning effect:

*Remembering the numbers and finding the little diamonds was, again, developing strategies, which sometimes didn’t work. So from that point of view, it was a more of a “strategy development” for me than just a straightforward blank “can you remember?”*

Gamification also appeared to help participants to stay engaged in repeated cognitive tasks, as some participants competed to improve their own previous score. In line with their reported motivations (theme 1), some participants self-reported benefits of regular cognitive testing such as boosted self-esteem, improved subjective cognitive functioning, and enjoyment, which may have supported prolonged engagement:

*Well, it made me feel very good when I found towards the end, I was getting better at it. I enjoyed it*.

However, repeating the same study tasks over time could also lead to fatigue and negative affect. Cognitive tasks could highlight participants’ cognitive concerns, meaning they became more frustrated, anxious, or disheartened with greater exposure to the tasks:

*Towards the end of the week, I was getting stressed with it. I think because it starts to put in your mind that maybe you are going “doolally.” Maybe at the beginning you’re a lot more aware of the numbers and you’re concentrating harder*.

Tasks that became progressively difficult appeared to prompt disengagement, even in participants who were initially enthusiastic about the tasks. Feeling demotivated may have meant that participants started to put in less effort or performed worse over time, which could impact the validity of high-frequency remote cognitive assessments:

*After a while, I kind of felt no matter how hard I tried, I just couldn’t...I didn’t exactly give up, but I certainly didn’t try as hard*.

While participants understood the rationale behind repeating the same series of cognitive tasks to enable comparison over time, the lack of variety meant that some participants transitioned from finding the study tasks engaging and enjoyable to tedious:

*I found it a chore doing them after the novelty had worn off, yeah. First, I thought, “Oh yes, I like puzzles.” And then I thought, “no, not this puzzle.”*

While repeated exposure to the same tasks could therefore create opportunities for learning and adaptation and participants understood why tasks were repeated, too much repetition of tasks, particularly cognitive tasks, may become a barrier to continued study participation or acceptability.

### Theme 4: Adherence, Accuracy, and Getting It Right

*I didn’t want to mess it up*.

#### Theme Overview

Participants were motivated to complete tasks as they had been instructed to and, for the most part, attempted to adhere to instructions on how and when to complete study tasks to the best of their ability. Perceptions of suboptimal performance on a task could contribute to negative affect including guilt, frustration, and anxiety. Social support helped to provide reassurance (theme 5).

#### “Am I Ruining the Study?”: Following the Protocol and Meeting Expectations

Participants took considerable efforts to complete the study tasks as instructed. When things went wrong or were difficult, some participants experienced negative affect including feeling guilty, embarrassed, or that they had failed in some way:

*I felt bad because I could not keep the headband on*.

Participants also found it more challenging to engage in tasks they subjectively felt they were not performing well on, including the cognitive tasks:

*It’s very frustrating. There’s nothing about the way you did it, or the methodology, or anything like that. That’s the problem; it’s just that sense of failure because you know you can't do them*.

Being unable to meet study requirements due to conflicting commitments was also stressful for participants, who wanted to follow the study protocol as closely as possible:

*It made me feel sort of a little bit kind of guilty, because I’m a conscientious person. [...] And then I thought, “Oh no, am I ruining the study because I’ve done this late?*”

Some participants found creative solutions to continue with study tasks when experiencing discomfort or when they had concerns around data quality. For example, participants reported using wires, bandages, caps, and gloves over the pulse oximeter or EEG headband to keep them in place, demonstrating how participants attempted to address concerns independently and through experience or trial and error, sometimes without consulting the research team:

*The Velcro pads were useless. I had to secure the headband using wire to connect the pads*.

However, support was often sought from the research team and relatives and is explored more in theme 5.

#### “I Couldn’t Really Get How It Was Supposed to Work”: Supporting Memory and Confidence Through Clear, Accessible Study Design

Participants with MCI or dementia were often concerned, particularly at the start of the study, whether their cognitive impairment and particularly their memory might impact participation. However, most participants remembered to complete tasks [33], either independently or following reminders sent by the research team:

*It was just, sort of, a slight kind of apprehension about being able to remember the actual tasks, but actually it didn’t, it wasn’t too bad*.

Having printed instructions reassured participants, allowed them to problem-solve whenever they needed it, and reduced reliance on memory. In contrast, on-screen instructions at the start of each set of the unsupervised cognitive tasks, which disappeared during the task, were less well-received. Not being able to remember the instructions when engaging in a remote cognitive task could affect task performance (eg, clicking in the wrong place during the choice reaction time task) and trigger disengagement:

*Well, you don’t then get access back to the instructions. So, you just get fed up*.

Verbal and written instructions could also be better complemented by visual or practical demonstrations. Participants were talked through and shown how to use the study kit at the start and were offered a call with a researcher before the intensive week. However, opportunities to physically practice tasks in the presence of the research team were recommended to build confidence in novel tasks:

*Despite having the instructions and everything, I would have found it helpful if...you showed me exactly how you did it, and I did it at the same time*.

#### “Being Able to Hazard a Guess”: Challenges in Accurately Capturing Sleep

Participants felt that recording sleep at home was more comfortable and that RMTs could provide a more accurate representation of their usual sleep than sleeping in a hospital or sleep laboratory:

*All I can say is, if I had had to do this study going into a room in a hospital, or some sort of clinical setting, I cannot imagine getting any sleep at all. So even though the quality of my sleep often wasn’t great, that wasn’t to do with my environment*.

However, concerns about devices working as intended and accurately recording sleep data were prevalent, particularly where participants had subjective sleep disturbance. One participant shared that they were skeptical about whether the devices could reliably track their sleep patterns because of nighttime movements:

*I tossed and turned too much. I don’t know whether the headband even managed to gather any data*.

Participants found it difficult to accurately self-report sleep metrics for their sleep diary and were aware that their responses might not align with the RMTs. Participants often used external cues, such as what television program had been on when they went to bed and what their partner thought, and internal cues, such as how rested and awake they felt, to help them estimate nighttime sleep duration, quality, and awakenings. Trying to complete a sleep diary was challenging due to issues with memory recall and being able to estimate the duration of time spent awake during the night:

*Did it take me 5 minutes to go to sleep, or did it take me 30 minutes? It’s all a bit hit and miss*.

Observer effects were also identified as a possible confound to accurately measuring sleep. For example, wearables that are uncomfortable could be a physical impediment to sleep (theme 2), while asking participants to reflect daily on their sleep could create greater self-awareness or anxiety around sleep:

*If I’m thinking about it too much, maybe I would stay awake more?*

### Theme 5: Social Support as a Facilitator and a Barrier

*A bit of human contact*.

#### Theme Overview

Participants had varying support needs, preferences, and expectations from both the research team and the relatives who provided informal study support. Support generally fell into categories: technical support (eg, to set up mobile apps, enter data, and join video calls) and cognitive and emotional support (eg, reminders on how to complete study tasks and motivation). Face-to-face contact and responsive communication with the research team were valued highly and supported continued engagement in the study.

#### “She Tends to Stand in for Me”: Technical Support and Troubleshooting

Someone at home, typically a partner or a relative, provided technical study support for many participants, including some controls with no cognitive impairment. The amount of support needed or provided varied substantially from support with initial setup and familiarization with novel apps to essential daily support (eg, completing digital sleep diaries):

*My wife is very technically clued up, and she tends to stand in for me*.

Participants also routinely contacted the research team for technical support as needed and to report issues. Confirming successful data transfer and data quality was reassuring and provided a sense of security and connection back to the research team. Participants reflected specifically on the speed and reliability of communication with the research team, and how email communication had an additional benefit of providing a record of the conversation for participants to look back on if they encountered the problem again:

*Knowing that if I had any queries, I could send you an email, and within a day or two, I got an answer or a chance to ask another question was good, that you had that level of not having to wait a long time if there was. Going through it when we met on the first day, and then by the time you come to use it, you kind of pretty much forgotten where to plug it in and what the button does, so it was quite good to have a refresher on that as well*.

#### “What Time Do We Go to Bed?”: Cognitive Support and the Study Partner

Partners and relatives also often helped with cognitive support through reminding participants to complete tasks and helped to answer the subjective questions on sleep, such as what time they woke up or went to bed:

*The main help I had from my wife saying, “You know what time, what time do we go to bed?” And “what time do you roughly think we went to sleep?*”

However, several participants, including those with MCI and dementia, did not have any support at home or chose to engage in the study completely independently and had no or few issues:

*I think I got on OK for myself*.

Furthermore, requiring a study partner as part of the eligibility criteria was identified as a barrier to research participation. One participant with MCI shared how they had been ineligible for previous dementia research because they did not have someone available to act as a study partner and had wanted to be able to take part independently:

*I needed to have somebody with me...But yeah, it didn't, it didn't come to fruition, because I couldn't get anybody to join me*.

#### “A Bit of Face-to-Face”: The Researcher-Participant Relationship and Face-to-Face Interactions

While most contact with the research team occurred remotely via email or video calls, in-person interactions at the clinic or at participants’ homes (eg, at baseline or midway through the study to collect study equipment or review how to complete study tasks) were expected, valued, and helped participants to feel more included in the research study:

*It’s nice to have a bit of face-to-face and a bit of human contact*.

Face-to-face interactions and developing rapport helped with confidence and mitigated against study fatigue or disengagement:

*I think, had you not come [for a home visit], I wouldn’t have finished it. I wouldn’t have...Confidence is right. Having a short contact with yourself was helpful. I think it helped me*.

Overall, interactions with others were an important facilitator offering both practical and social support during study participation, while study partner requirements were a barrier.

### Theme 6: Reflections, Realities, and Uncertainties Around Sleep

*Is it just a lazy habit?*

#### Theme Overview

Participants generally perceived good sleep as important for overall and cognitive health but were often unsure how to achieve it. Subjective sleep quality was associated with factors such as snoring, dreaming, sleep duration, and nighttime awakenings.

#### “There’s a Lot of Talk”: Factors Influencing Subjective Sleep Quality

Total sleep duration, or getting “enough” sleep, was recognized as a key component of sleep quality by participants. Having approximately 8 hours of sleep was recognized as the target for adults, but this was not always considered to be achievable or common:

*There’s a lot of talk about ‘you should get sort of eight hours solid sleep a night’ and I don’t know anybody who does*.

Daytime napping was also raised as an important component of sleep among older adults. Some participants used napping as a strategy to manage energy levels throughout the day or avoid going to bed early, whereas others were concerned about the health consequences of taking daytime naps and would actively avoid napping despite daytime sleepiness:

*I try to force myself through the day without taking that middle of the afternoon or late afternoon nap*.

Snoring was also a concern regarding sleep disturbance. Snoring affected not only individuals but also their partners, sometimes leading to disrupted sleep, frustration, or changes in sleeping arrangements, highlighting the social as well as personal impact of sleep disturbances:

*The biggest issue with my sleep has always been my snoring*.

Vivid dreams were also discussed as a factor affecting sleep quality:

*I was having quite an intense dream, you know, quite vivid, and it drifted into reality the following day*.

Overall, participants shared a range of components that could shape their perception of a good night’s sleep, extending from meeting recommendations and comparing to how others sleep to its impact on daily and social functioning.

#### “It’s Very Hard to Get Up and Start the Day”: Perceived Consequences of Poor Sleep

Many participants shared how they felt both sleep and research investigating how sleep might impact health, cognition, and dementia were important:

*The connection between sleep and dementia is important...I was interested to see if my sleep quality affected how I managed the cognitive tests*.

Some participants noted that they typically felt worse and experienced worse cognition following poor sleep, such as experiencing brain fog, difficulty processing information, and lack of motivation:

*I’m interested, because I haven’t slept well for so many years, really. And sometimes I feel like I haven’t really slept at all. And it’s very hard to get up and start the day when you’ve had that*.

Efforts to improve sleep largely focused on lifestyle changes and sleep hygiene practices, such as sleep regularity, avoiding foods that might disturb sleep, and managing stress and emotions. However, participants were keen to seek advice about good sleeping practices and often were unsure about healthy sleeping practices:

*When you wake and you doze, I mean, should you get up and start your day after your five hours? Or is dozing useful? Or is it just a lazy habit?*

However, some participants doubted whether sleep had benefits:

*The only thing for me is that I didn’t perceive any benefit of good sleep*.

Overall, while some participants were aware of its importance or impacts, sleep was not universally recognized by the participants as a core component to cognitive or brain health.

## Discussion

### Principal Findings

This study analyzed the experiences of older adults with and without cognitive impairment in a research study involving multimodal remote monitoring of sleep and cognition. We identified 5 themes around barriers and facilitators for remote sleep and dementia research, which centered around perceptions of the study as helpful or beneficial to themselves or others (*perceived value as motivation*); requiring devices to be ergonomic and reliable (*trust and simplicity as cornerstones in user experience*); the importance of repetition, flexibility, and routine (*adjusting to study participation over time*); a drive to “do well” and provide good outcomes (*adherence, accuracy, and getting it right*); and the value of help from others (*social support as a facilitator and a barrier*). Overall, acceptance and engagement were high among older adults with and without cognitive impairment, which aligns with our feasibility findings [33]. However, we identified several opportunities for improvement in future research designs which could improve acceptability. We also identified a sixth theme, which highlighted a knowledge gap and interest in sleep hygiene and the potential value of sleep education in older adults (*reflections, realities, and uncertainties around sleep*).

Engagement in the study was primarily facilitated by participants’ extrinsic motivations to help others and themselves and intrinsic motivations of interest and enjoyment. Altruism and opportunity for personal benefit have previously been identified in cognitively healthy older adults engaging in dementia prevention trials [46] and patients and caregivers of people living with dementia [47]. Receiving incentives was also discussed, aligning with studies in which receiving financial reimbursement for enrollment [48], biomarker results [49], and feedback on cognitive performance [50] were used to encourage research participation and engagement. While real-time feedback during a trial is typically avoided to reduce potentially confounding results, payments or high-level personalized summaries for participants at the end of a trial might be incentivizing [46].

Research participation is time-consuming and interfering with meaningful activities or commitments may be a barrier to engagement [47]. In this study, convenience and flexibility facilitated integration of study tasks into daily routines and supported continued participation. Remote study tasks may help to reduce the time and effort involved in attending clinic visits; however, inflexible scheduling or inconvenient timing of remote data collection may result in missing data or additional participant burden. For sleep and circadian research, it may be necessary to collect data at inconvenient times, such as around awakening, mealtimes, or bedtime. Researchers should carefully consider which tasks would benefit most from restricted time windows and allow greater flexibility for other variables where feasible. Greater use of passive monitoring devices, which continuously collect data without requiring active involvement from participants, may also help to increase convenience and reduce burden [51].

Social support encouraged continued engagement and fostered positive experiences for some participants, whereas others engaged in the study independently, highlighting the importance of individualized approaches to support. Mandatory study partner requirements may unnecessarily exclude capable individuals from research participation, particularly for cognitively unimpaired older adults or individuals with MCI [20]. Despite this, study partners are often considered a requirement in prodromal dementia research [20,52]. Study partners in dementia research can offer invaluable support, facilitation, and encouragement, as well as act as informants to help corroborate on symptoms experienced by patients. However, supporting patients with MCI or dementia to engage in research can also have both practical (eg, logistics of attending visits or calls with researchers) and emotional (eg, acknowledging cognitive decline) burden on the study partner [53], which can prohibit carers, and therefore patients, enrolling in dementia research [47]. For an inclusive and acceptable approach to dementia research, our results highlight the value in flexibility around study partner involvement—benefiting from their support and insight when it is available, without unnecessarily excluding patients who could participate independently.

Regular and responsive communication with a researcher supported troubleshooting and data collection and provided opportunities for meaningful social interactions. Positive interactions and developing trust and rapport between participants and researchers have been recognized as important components in encouraging participant retention, including in dementia research [54,55]. Maintaining contact with research participants requires additional time and effort from research delivery staff; however, there is often more funding and delivery time allocated to recruitment than retention during trials [56]. Allocating sufficient time and flexibility for participant support and follow-up during longitudinal research could meaningfully support engagement and retention of participants traditionally underserved in research [57] and may be particularly valuable as trials become more decentralized.

Participants evaluated study devices and tasks based on perceptions of comfort, usability, familiarity, security, and intrusiveness. Technical reliability was important for maintaining participant confidence, and simple, integrated platforms could help to improve user experience. Balancing user comfort and functionality is a key feature common to ergonomic design, although RMTs and smart technology focusing on health are often designed to appeal to adults with already healthy lifestyles aiming to optimize and track their habits [58], rather than older adults with cognitive impairment. Simple instructions that remain on-screen, lower reliance on fine motor skills (such as the use of voice-assisted apps), being minimally intrusive to daily routines, and incorporating some variety to maintain interest may be particularly helpful for older adults with cognitive impairment or medical comorbidities. More interactive study tasks can mean more issues or queries, which has led some researchers to encourage moving toward passive RMTs [20]. However, removing all active study tasks may also remove some of the participant satisfaction and engagement. Interactions with RMTs may create opportunities for cognitive stimulation and help foster a sense of meaningful and deliberate contribution to the study [59]. Balancing passive and active monitoring to ensure sufficient variation to maintain interest and appeal to participants with different preferences may be desirable.

Participants’ user experiences and attitudes often changed across the course of the study. Shifts toward positive user experience were observed as participants became more familiar and comfortable with the novel RMTs and successfully integrated tasks into their day by developing routines. However, repetition could be both a barrier and facilitator to continued engagement. For example, while some participants gamified the repeated cognitive challenges and saw them as opportunities to improve their scores, for others this repetition was emotionally distressing or fatiguing, particularly if they felt that they were not doing well. Most participants continued the tasks in our study [33], despite frustration when faced with challenges. In longitudinal aging studies using repeated cognitive testing, attrition has been linked to lower cognitive and functional ability [51]. Participants who reported feeling cognitively disengaged may have eventually stopped completing the tasks altogether. Incorporating variation in cognitive tests and minimizing the number of tasks that are taken to failure may help to reduce study fatigue and sustain engagement.

Finally, participants reported difficulty in self-reporting sleep outcomes, particularly sleep latency and wake after sleep onset. We observed discrepancies between subjective and objective sleep estimates in the larger cohort, particularly for cognitively impaired older adults who tended to underestimate their sleep disturbance [8]. Together, our findings support the growing literature that subjective sleep estimation is challenging and should not be used as a proxy for objective sleep in this population [60-62].

### Limitations

Remote research using various virtual data collection strategies (videoconferencing, online questionnaires, and virtual focus groups) to decentralize dementia research may show promise in diversifying trial populations [63,64]. The study enrolled participants from 1 geographical area who were predominantly White British and male, which may limit generalizability to broader, more diverse populations. Cultural and sex differences may influence the acceptability of research specifically using wearable or digital health technologies. For example, individuals who are White and speak English often have more access and are more likely to use digital health technologies [65,66] and have fewer concerns about whether research is trustworthy, invasive, or beneficial to their community [67]. Additionally, men tend to report higher technological confidence [68]. Lower levels of acceptability or different barriers may have been identified in a more diverse sample.

Self-selection bias may also have influenced the acceptability. Participants in RESTED were generally familiar with technology, albeit not most of the devices used in this study. Baseline digital literacy can be a strong predictor of engagement with RMTs, with less digitally experienced older adults showing higher dropout rates and reporting greater anxiety when using multiple digital platforms [69]. Additional support strategies or simplified interfaces may be necessary to ensure equitable access to remote sleep monitoring for all older adults, regardless of their prior technology experience [70]. Moreover, as only 1 participant withdrew from the study and they declined an interview, the analysis focused on participants who engaged throughout the study. Interviewing eligible patients who did not participate in the study would increase understanding barriers to participation [47].

A strength of our study was the duration, as it examined acceptability over 8 weeks. However, our results may not generalize to more extended monitoring periods over months or years. Future studies should examine how to optimize acceptability of technology-based research over more prolonged periods and as cognitive impairment progresses [69], as RMTs may be particularly suited to longitudinal cohort studies examining disease progression.

Finally, future research could examine whether there are any systematic differences among individuals at different stages of cognitive impairment in terms of preferences and acceptability, as different levels of engagement can be influenced by cognitive status [30]. While the quantitative feasibility paper for the RESTED study identified high levels of adherence and good data quality across the AD, LBD, and cognitively healthy controls [33], acceptability may differ between cognitively unimpaired versus cognitively impaired older adults.

### Conclusions

To our knowledge, this is the first qualitative study examining the acceptability and participant experiences of longitudinal sleep and dementia research in older adults. Our study demonstrates that older adults with MCI and dementia and cognitively unimpaired older adults are motivated and able to engage meaningfully with research using remote study tasks and digital health technology when provided with appropriate devices, support, and flexibility. Understanding the specific barriers and facilitators identified in our study will enable researchers to design more inclusive and effective remote monitoring protocols that accommodate the needs of older adults with cognitive impairment, advance our understanding of the relationship between sleep and dementia, and support future patient-centered interventional trial design.

## Supplementary material

10.2196/88094 Multimedia Appendix 1 Standards for Reporting Qualitative Research (SRQR) checklist, interview topic guide, questionnaires, and further information on how themes mapped to the COM-B and UTAUT-2 model.

## Abbreviations

AD: Alzheimer disease
COM-B: Capability, Opportunity, Motivation–Behavior
EEG: electroencephalography
LBD: Lewy body disease
MCI: mild cognitive impairment
REM: rapid eye movement
RESTED: Remote Evaluation of Sleep to Enhance Understanding of Early Dementia
RMT: remote monitoring technology
UTAUT-2: Unified Theory of Acceptance and Use of Technology
